# Reproducibility and positioning sensitivity of CT beam width measurements using a pencil ionization chamber and radiopaque mask

**DOI:** 10.1002/acm2.70753

**Published:** 2026-08-24

**Authors:** Morgan Aire, Hunter C. Meyer, Krystal Kirby

**Affiliations:** ^1^ Department of Physics and Astronomy Louisiana State University Baton Rouge Louisiana USA; ^2^ Department of Radiation Oncology Mary Bird Perkins Cancer Center Baton Rouge Louisiana USA

**Keywords:** beam width, computed tomography, ionization chamber, quality assurance, reproducibility

## Abstract

**Purpose:**

To evaluate the reproducibility and precision of CT beam width measurements using a pencil ionization chamber and radiopaque mask and to assess the robustness of the technique against clinically realistic setup errors in the superior‐inferior (SI) and anterior‐posterior (AP) directions.

**Methods:**

Beam width was measured on a GE Discovery RT590 CT scanner at three nominal collimations (10, 15, 20 mm), three tube potentials (80, 100, 120 kV), and three tube currents (50, 100, 150 mA). Three repeated exposures per setting were acquired using a Radcal Accu‐Gold pencil ionization chamber with a radiopaque mask to calculate beam width. Coefficients of variation and Levene's test were used to test precision. To assess the robustness to setup error, the chamber was offset from the isocenter at 0, 1, 3, and 5 mm in the SI direction at 20‐mm collimation across all kV/mA combinations and at 0 and 5 mm in the AP direction at 10‐ and 20‐mm collimation. A linear mixed‐effects model was used to test the sensitivity of beam width to SI setup errors across kV/mA combinations. Slopes of the overall deviance from the global mean vs offset were obtained for each combination with 95% confidence intervals, and pairwise slope differences were evaluated using a Tukey‐adjusted statistical test. Confidence intervals were used to assess AP setup errors at two different nominal collimations.

**Results:**

Across all kV/mA combinations, mean measured radiation beam width was 13.04, 16.90, and 20.60 mm for nominal 10‐, 15‐, and 20‐mm collimations, with inter‐protocol coefficients of variation of 0.57%, 0.48%, and 0.25%, respectively. Intra‐protocol standard deviations across triplicate exposures were 0.04 to 0.09 mm. SI offset slopes from 0 – 3 mm ranged from −0.07 to −0.12 mm (per mm of offset) with overlapping 95% confidence intervals. There was no significant interaction with kV/mA (*p* > 0.05). AP offsets of 5 mm produced beam‐width changes of 0.09 mm at 10 mm collimation and 0.15 mm at 20 mm collimation.

**Conclusion:**

The pencil ionization chamber and radiopaque mask technique produced highly reproducible CT beam width estimates for potentials up to 120 kV and at or above 50 mA that were independent of tube potential, tube current, and robust to clinically plausible setup inconsistencies.

## 1 INTRODUCTION

The radiation beam width along the longitudinal (*z*) axis is a fundamental parameter of CT scanners, governing the irradiated z‐extent per rotation and contributing to the volume CT dose index calculation. Accurate characterization of beam width is therefore essential for reliable dose reporting and for verifying that scanner performance remains within manufacturer specifications. As a result, the American College of Radiology (ACR) CT Quality Control Manual requires annual measurement of the beam width during quality assurance testing of accredited CT machines.^1^ The manual states that manufacturer specifications can be used, and if those are not available, recommends that the beam width fall within 3 mm or 30% of the specified beam width value, whichever is greater.

Several techniques have been used to measure CT beam width and have been described in AAPM Task Group 233, including radiographic and radiochromic films, computed‐radiography (CR) imaging plates, and pencil ionization chamber readings.^2^ Each method has trade‐offs in cost, throughput, and operator dependence.^3^, ^4^ Film‐based methods are particularly sensitive to processing variability, development time, and post‐irradiation darkening, which limits their precision.^5^, ^6^, ^7^

Recently, Al‐Senan et al.^8^ described a method that uses a 100 mm pencil ionization chamber together with a calibrated cylindrical radiopaque mask to determine the beam width. This method reported root‐mean‐square agreement of less than 0.5 mm against film and CR references across nominal collimations from 1 to 40 mm. The technique used a single tube potential of 80 kVp, noting the low kV minimized collimator‐induced leakage and transmission of the primary beam through the mask wall. Because clinical CT quality control is performed at institution‐specific tube potentials that often exceed 80 kVp, it remains unknown whether this technique retains its accuracy and reproducibility across the broader kV range used in practice. It is also unknown how sensitive this technique is to small isocenter setup errors that are common in routine QA.

The purpose of this study was to evaluate the reproducibility of this beam‐width measurement method across clinically relevant kV/mA combinations and to assess its sensitivity to small positioning errors in order to establish its suitability for routine quality control.

## 2 METHODS

### 2.1 Experimental setup and beam‐width calculation

Measurements were performed on a GE Discovery RT590 CT scanner (GE HealthCare, Chicago, IL, USA). Exposures were acquired using a Radcal Accu‐Gold+ Touch digitizer/electrometer and 100 mm Radcal pencil ionization chamber (Radcal Corporation, Monrovia, CA, USA).

The experimental technique was implemented as described in Al‐Senan et al.^8^ The ionization chamber was placed along the longitudinal (*z*) axis with the center of the sensitive region aligned at the isocenter using the scanner lasers. A cylindrical radiopaque tungsten mask from the Radcal CT Beam Width Tool Kit of length, $L_{eff}=10.05\;\mathrm{mm}$, inner diameter 9.4mm, and outer diameter 15.4mm, as defined by the manufacturer, was placed over the chamber and centered at isocenter.

An identical axial scan was acquired with ($X_{masked}$) and without ($X_{unmasked}$) the mask at the different kV/mA combinations at the largest available nominal collimation (20 mm). An exposure per unit length calibration factor (CF) was defined by:

(1) $$CF=\frac{\left(X_{unmasked}-X_{masked}\right)}{L_{eff}}$$

The radiation beam width, $W_{b}$, was defined as the quotient of the calibration factor to the specified exposure for a given nominal collimation, $X_{coll}$:

(2) $$W_{b}=\frac{X_{coll}}{CF}$$

With the CF determined at the calibration condition, the measured beam width at any other nominal collimation can be calculated without the mask at the same kV, mAs, pre‐patient filtration, and location.

### 2.2 Reproducibility across kV/mA combinations

Beam width measurements were acquired at nominal collimations of 10 mm (8 × 1.25 mm), 15 mm (4 × 3.75 mm), and 20 mm (8 × 2.5 mm) for all combinations of tube potentials (80, 100, 120 kV) and three tube currents (50, 100, 150 mA), yielding 27 protocol combinations. The three largest available nominal collimations were selected because they are the collimations used clinically at our institution and are expected to be more sensitive to small off‐axis errors. Tube currents of 10, 20, 30, and 40 mA were evaluated but did not produce consistent readings across all tube potentials and were therefore excluded. The minimum tube current yielding consistent, detectable readings across all tube potentials was 50 mA, which was therefore set as the lower bound of the tube current range. Each protocol was repeated three times. For each protocol, the mean, standard deviation, standard error, and 95% confidence intervals of the three measurements were computed. Across protocols, the inter‐protocol mean and coefficient of variation were computed for each nominal collimation. Levene's test for homogeneity of variance was performed across kV/mA groups within each nominal collimation to assess whether measurement precision was equivalent across acquisition parameters.

### 2.3 Sensitivity to setup error

To evaluate the robustness of the method to common setup errors, two experiments were performed at 20 mm nominal collimation. Setup errors were introduced by adjusting the table position. In the SI experiment, the chamber was longitudinally offset from isocenter by 0, 1, 3, and 5 mm for each of the nine kV/mA combinations, with three repeated exposures per condition. A 5 mm offset was selected as the largest value because it is the value at which the alignment lasers touch the edge of the tungsten ring, and any further misalignment would not have the lasers touching any part of the measurement setup. The chamber was offset in the superior direction with the assumption that any change in dose‐profile symmetry about isocenter due to the heel effect is negligible at this distance,^9^, ^10^ consistent with prior reports that heel‐effect asymmetry is negligible for narrow x‐ray beam widths and becomes appreciable only for wide cone‐beam geometries (> 100 mm collimation). A linear mixed‐effects model was used to regress beam width deviation from the global mean, specified as the mean across nominal‐position measurements. The per‐combination slopes (mm of deviation per mm of offset) were extracted with 95% confidence intervals (CIs), and pairwise differences between slopes were tested using Tukey‐adjusted *p*‐values. Statistical significance was defined as *p* < 0.05 for all comparisons.

In the anterior‐posterior (AP) experiment, the chamber was offset from the isocenter in the anterior direction by 0 and 5 mm for both 10‐ and 20‐mm nominal combinations, at 120 kV and 150 mA. A 5 mm offset was selected as that is the value at which the alignment lasers no longer touch the ionization chamber or mask. This therefore represents a realistic upper bound on positioning error in clinical practice. A single kV/mA combination (120 kV/150 mA) was selected based on the results of Section 2.2, which demonstrated that beam width is effectively independent of tube potential and current, making a full factorial AP experiment redundant. The change in measured beam width between the 0 and 5 mm AP offsets was tabulated for each collimation with confidence intervals.

All statistical analyses were performed in R. Claude (Claude Sonnet 4.6, Anthropic) was used for statistical R code debugging and manuscript editing.

## 3 RESULTS

### 3.1 Reproducibility across kV/mA combinations

Inter‐protocol mean beam width values are shown in Table 1. Nominal dose efficiency values were obtained from the scanner specifications. Coefficients of variation across the 27 kV/mA combinations were below 0.6% at all three nominal widths, and the inter‐protocol standard deviation was less than or equal to 0.08 mm in every case. Intra‐protocol standard deviations across the repeated exposures ranged from 0.04 to 0.09 mm, with standard errors below 0.06 mm. *F*‐values from Levene's test revealed no significant difference in measurement variance across kV/mA combinations within any collimation width (*p* > 0.90), indicating that the precision of beam width measurements is stable and equivalent regardless of kV/mA combination.

**TABLE 1:** Summary of Beam width measurements across all kV and mA combinations.

Nominal collimation (mm)	Detector configuration	Nominal dose efficiency (%)	Mean beam width (mm)	Standard deviation (mm)	Coefficient of variation (%)	*F*‐value
10	8 × 1.25 mm	79.93%	13.04	0.07	0.57	0.12
15	4 × 3.75 mm	93.15%	16.90	0.08	0.48	0.17
20	8 × 2.5 mm	98.93%	20.60	0.05	0.25	0.08

**FIGURE 1:**
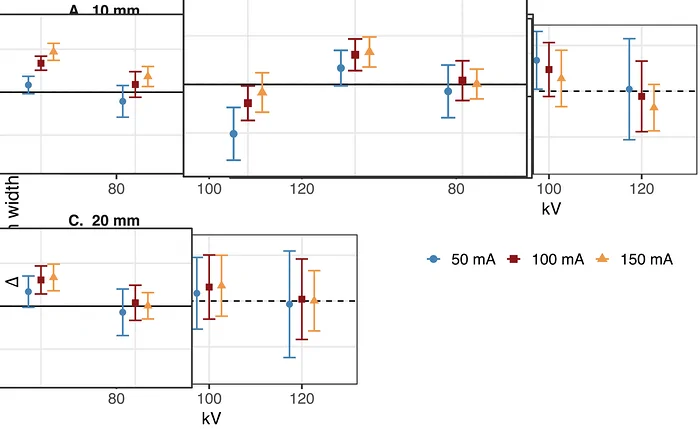
Measured deviation from scanner‐specific mean beam width as a function of tube potential for nominal (A) 10, (B) 15, and (C) 20 mm collimations, using a pencil ionization chamber and radiopaque mask technique. Each point represents the mean of three measurements with error bars indicating the 95% confidence interval. The dashed horizontal line at zero indicates no difference from the collimation‐specific mean. Deviations above and below represent over‐ and underestimation relative to that mean.

The deviation of each kV/mA‐specific mean from the overall collimation‐specific mean is plotted in Figure 1. All deviations were within 0.15 mm of the collimation‐specific mean. No systematic dependence on tube potential or tube current was apparent. The largest absolute deviations occurred at 80 kV/50 mA at the 10 mm collimation (−0.14 mm).

### 3.2 Sensitivity to SI offset

The estimated slope of beam width deviation versus SI offset is shown in Table 2 for each kV/mA combination at 20 mm collimation over the valid measurement range (0 – 3 mm). The slope represents the rate of change in measured beam width per millimeter of SI displacement from isocenter, estimated from a linear mixed‐effects model, where a slope of zero would indicate complete insensitivity to SI offset. All nine slopes were small and slightly negative, ranging from −0.068 to −0.118 mm of beam width change per mm of SI offset, with overlapping 95% CIs. A pairwise comparison of slopes using the Tukey‐adjusted p‐values found no statistically significant interactions (all p > 0.05), indicating that the sensitivity of the measurement to SI setup error did not depend on tube potential or tube current. Over this range, even a 3 mm SI offset produced a beam‐width change of less than 0.4 mm, a value much smaller than the ACR tolerance of 3 mm, indicating that it is unlikely to push a measurement out of tolerance.

**TABLE 2:** Sensitivity of measured beam width to SI setup error at 20 mm collimation.

kV	mA	Slope (mm/mm)	95% CI
80	50	−0.068	[−0.089, −0.047]
80	100	−0.072	[−0.103, −0.042]
80	150	−0.071	[−0.103, −0.040]
100	50	−0.090	[−0.127, −0.052]
100	100	−0.089	[−0.123, −0.055]
100	150	−0.096	[−0.135, −0.058]
120	50	−0.115	[−0.145, −0.085]
120	100	−0.110	[−0.148, −0.071]
120	150	−0.118	[−0.152, −0.083]

At a 5 mm SI offset, the alignment laser intersects the edge of the tungsten ring, placing the chamber outside the valid measurement geometry. At this displacement, the measured beam width increased abruptly by approximately 1.4 mm across all kV/mA combinations (mean +1.43 mm), reflecting breakdown of the calibration geometry rather than a continuation of the gradual trend observed at smaller offsets. Because a 5 mm offset produces a visually obvious laser misalignment, such an error would be readily detected and corrected before measurement in routine practice.

### 3.3 Sensitivity to AP offset

AP offset results at 120 kV and 150 mA are summarized in Table 3. A 5 mm AP shift produced a beam width change of 0.09 mm at 10 mm collimation and 0.15 mm at 20 mm collimation. Both changes are an order of magnitude smaller than the ACR tolerance, indicating that AP positioning errors of clinically plausible magnitude do not meaningfully degrade the measurement or cause it to be out of tolerance. The slightly larger change in beam width at 20 mm collimation is consistent with the wider chamber‐projection geometry at the larger nominal width.

**TABLE 3:** Sensitivity of measured beam width to AP setup error at 120 kV/150 mA.

Nominal beam collimation (mm)	AP offset (mm)	Mean measured beam width (mm)	Range (mm)	Δ beam width (mm)
10	0	12.75	[12.75, 12.76]	0.09
	5	12.66	[12.62, 12.74]
20	0	20.77	[20.76, 20.78]	0.15
	5	20.62	[20.56, 20.75]

The change in beam width is well within the 30%‐ and 3‐mm tolerance at both collimation widths, indicating that AP positioning errors of this magnitude may not produce meaningful differences.

## 4 DISCUSSION

In this study, we measured the reproducibility of radiation beam width measurements using a pencil ionization chamber for different kVp/mA combinations and measured the sensitivity to setup errors. We found that beam width measurements were highly consistent across kV/mA combinations under nominal positioning. Second, sensitivity to SI setup error was uniform across all acquisition parameters. Finally, AP positioning errors of clinically plausible magnitude did not produce statistically significant changes in beam width.

Under perfect positioning conditions, all measured beam widths fell within 0.15 mm of their respective collimation‐specific mean across all kV/mA combinations and all three nominal collimation widths. Levene's tests for homogeneity of variance demonstrated equivalent measurement precision, indicating that the choice of tube potential and current does not influence the precision of beam width measurement in this scanner. These findings support the use of any of the tested kV/mA combinations up to 120 kV and at or above 50 mA for routine quality control on this scanner. This study was conducted on a single scanner (GE Discovery RT590). As with the original method by Al‐Senan et al.,^8^ which was similarly limited to GE and Philips platforms, this narrows evaluation of accuracy, reproducibility, and sensitivity to the tested hardware, and findings may not generalize to other manufactures, other GE Discovery models, or scanners with maximum collimations larger than 20 mm. Differences in focal‐spot size or pre‐patient geometry on other systems may also be more sensitive to kV/mA combinations or positional offsets

Over the range of clinically plausible, non‐obvious setup errors (0–3 mm), all tested combinations exhibited small negative slopes of beam‐width deviation versus SI offset, though there were no statistically significant differences in slope between any kV/mA combinations. This indicates that although the measurement exhibits modest sensitivity to SI positioning within the valid range, this sensitivity is uniform across acquisition parameters. The uniformity of the response is consistent with a geometric rather than energy‐dependent explanation, supporting the conclusion that the method's behavior under realistic setup perturbation is predictable and independent of acquisition parameters. Because the ACR exclusion criterion is 3 mm or 30%, whichever is greater, clinically realistic SI positioning errors do not constitute a beam‐width QA failure on this system. At larger offsets, the measurement geometry breaks down, and the measured width increases sharply. However, such gross misalignment is self‐evident and would be corrected before acquisition.

The change in beam width for AP was well within the 30%‐ and 3‐mm tolerance, indicating that the observed changes are minimal. The slightly larger absolute change observed at 20 mm collimation is consistent with the wider chamber‐projection geometry at larger nominal collimations. Notably, although a 5 mm AP offset is also a visually apparent setup error, it produced no meaningful change in measured beam width, in contrast to the 5 mm SI offset. This asymmetry reflects the geometry of the measurement. An SI displacement moves the chamber along the sensitive axis and beyond the z‐extent of the mask, corrupting the calibration. An AP displacement moves the chamber perpendicular to its axis through a broad, approximately uniform region of the beam, leaving the sampled *z*‐profile essentially unchanged.

The AP offset experiment was conducted at a single tube potential and current combination. While this represents a common clinical output condition, it cannot exclude protocol‐dependent variability in AP sensitivity across the full kV/mA matrix. A complete factorial AP offset experiment, analogous to the SI analysis presented here, would determine whether AP sensitivity is modulated by tube potential or current, an interaction that the present single‐combination design cannot resolve.

The absolute accuracy of the mask‐and‐chamber method was not re‐evaluated in this study, as it has been established previously on comparable equipment.^8^ Validation of absolute accuracy is an opportunity for future work. Construction of a purpose‐built positioning stand with calibrated angular and lateral markings would enable a rigorous quantification of the sensitivity of the method to angular misalignment and right‐left lateral offsets.

## 5 CONCLUSION

The pencil ionization chamber and radiopaque mask technique produced highly reproducible CT beam‐width measurements for potentials up to 120 kV and at or above 50 mA that were independent of tube potential and tube current and robust to clinically realistic SI and AP setup errors. Measurement sensitivity to SI offset was uniform across all tested acquisition parameters, and AP offsets of clinically plausible magnitude produced no meaningful change in measured beam width.

## AUTHOR CONTRIBUTIONS

**Morgan Aire**: Data acquisition; interpretation; analysis; figure and table creation; writing and drafting work. **Hunter C. Meyer**: Data acquisition; interpretation; analysis; writing and drafting work. **Krystal Kirby**: Supervision; data interpretation and analysis. All authors contributed to the manuscript preparation and final editing and review.

## CONFLICT OF INTEREST STATEMENT

The authors declare no conflicts of interest.
